# Hidden Adiposity Beyond BMI: Body Composition and Lifestyle Profiles Among Sport Sciences Students at Universidad Técnica del Norte, Ecuador

**DOI:** 10.3390/healthcare14213656

**Published:** 2026-10-28

**Authors:** Pablo Buitrón-Jácome, Jorge-Elías Rivadeneira, Elmer Meneses-Salazar, Alexandra del Carmen Mina Páez, Marco Hernández-Martínez

**Affiliations:** 1 Society and Physical Activity Research Group (GISAF), Faculty of Education, Science and Technology, Universidad Técnica del Norte, Ibarra 100105, Ecuador; jerivadeneira@utn.edu.ec (J.-E.R.); eomeneses@utn.edu.ec (E.M.-S.); acmina@utn.edu.ec (A.d.C.M.P.); 2 Ministerio de Educación, Deporte y Cultura, Ibarra 100105, Ecuador; marco.hernandez@docentes.educacion.edu.ec

**Keywords:** hidden adiposity, body composition, lifestyle profiles, university students, sport sciences

## Abstract

**Background/Objectives**: Body mass index (BMI) is widely used to assess weight status but does not directly characterize body composition. Lifestyle assessment may provide complementary behavioral information. This study examined BMI–body-fat classification discordance and compared body-composition and lifestyle profiles across operational adiposity phenotypes among sport sciences students at Universidad Técnica del Norte, Ecuador. **Methods**: An analytical cross-sectional study was conducted in a non-probability convenience sample of 303 students enrolled in two sport sciences degree programs. Body composition was estimated by bioelectrical impedance analysis using the InBody 270, and health-promoting lifestyle behaviors were assessed using a 52-item Spanish-language adaptation of the Health-Promoting Lifestyle Profile II (HPLP-II). Elevated body fat was defined using sex-specific operational criteria (>20% in men and >28% in women). BMI classification performance and differences in body-composition and lifestyle characteristics across phenotypes were analyzed. **Results**: Elevated body fat was identified in 53.5% of participants, and 19.5% met the operational criterion for hidden adiposity. Among participants with normal BMI, 32.1% had elevated body fat. Relative to the operational BIA-based criterion, BMI showed 63.6% classification agreement for elevated body fat and a 36.4% false-negative rate. Participants with hidden adiposity showed lower muscle-related indicators and lower scores in physical activity, health responsibility, and stress management, although lifestyle-related effect sizes were generally small. **Conclusions**: Combining BMI with BIA-derived body-composition estimates revealed classification discordance not captured by BMI alone. Selected HPLP-II domains also differed between phenotypes, although effect sizes were small. These findings describe an operational body-composition phenotype and should not be interpreted as evidence of clinical obesity or metabolic disease.

## 1. Introduction

Higher education constitutes a complex social environment in which professional training, the reorganization of daily routines, and the progressive development of personal autonomy converge. Within this context, students face academic, economic, and interpersonal demands that may significantly affect their eating habits, sleep, physical activity, and stress management. These practices do not develop in isolation but are shaped by characteristics of the university environment, including class schedules, commuting demands, support networks, perceptions of inclusion, and opportunities for participation. Recent evidence indicates that lifestyle-related behaviors are associated with psychological distress and well-being, whereas experiences related to social integration, security, and belonging are also relevant to students’ adaptation within the university environment [1,2].

The transition to university life generally involves greater autonomy in food-related decision-making; however, this autonomy is often constrained by limited time, financial resources, and food availability. Consequently, university students’ eating habits result from a complex interaction among individual, social, and environmental factors and cannot be explained solely by nutritional knowledge or individual willpower [3,4]. Similarly, physical activity and sleep are influenced by academic workload, prolonged use of electronic devices, irregular schedules, and reductions in unstructured daily movement. From this perspective, lifestyle may be understood as the outcome of the interaction between individual decisions and the opportunities and constraints imposed by the university environment.

The Health-Promoting Lifestyle Profile II (HPLP-II) organizes health-promoting behaviors into six domains: physical activity, nutrition, health responsibility, stress management, interpersonal relations, and spiritual growth [5]. In the Mexican adaptation used in the present study, the domain corresponding to spiritual growth was labeled positive psychology [6]. This six-domain framework permits a multidimensional description of health-promoting behaviors. Research involving university populations has shown that physical activity is associated with sleep and other components of well-being, although the direction and magnitude of these associations depend on the context, study design, and methods used to measure behavior [7]. Accordingly, examining lifestyle domains jointly may provide a broader behavioral description than assessing individual behaviors in isolation.

Nevertheless, the assessment of weight status in student populations continues to rely frequently on body mass index (BMI). Its use has become widespread because of its simple calculation, low cost, and usefulness for describing weight distributions at the population level. However, limitations arise when the relationship between weight and height is interpreted as a direct measure of adiposity at the individual level, potentially resulting in misclassification of adiposity. BMI does not distinguish fat mass from muscle mass, does not describe the distribution of adipose tissue, and may classify individuals with different body compositions within the same category. A systematic review and meta-analysis found that BMI has high specificity but limited sensitivity for identifying excess body fat, indicating that some individuals with elevated adiposity may remain within the normal BMI category [8].

Current debate increasingly emphasizes defining more precisely the scope of the information provided by BMI rather than replacing it. In this regard, the Lancet Diabetes & Endocrinology Commission on the definition and diagnostic criteria of clinical obesity proposed retaining BMI as a screening and population-surveillance tool while emphasizing that it should not be used in isolation to confirm excess adiposity or diagnose clinical obesity. At the individual level, BMI interpretation should be complemented by body-composition assessments or additional anthropometric indicators. Furthermore, the diagnosis of clinical obesity requires confirmed excess adiposity together with evidence of organ dysfunction or functional limitations attributable to excess adipose tissue. This distinction is particularly relevant in non-clinical studies of university populations, in which identifying an elevated body fat percentage may reveal body-composition patterns not captured by BMI, without establishing the presence of clinical obesity, metabolic disease, or increased individual cardiometabolic risk [9].

A specific manifestation of these limitations is the presence of individuals whose BMI falls within the normal range but who nevertheless have an elevated body fat percentage. This phenotype has been described as normal-weight obesity or elevated adiposity with normal BMI [10,11]. Some studies have associated this phenotype with lower fat-free mass, greater central adiposity, and less favorable cardiometabolic profiles [9,10]. However, definitions vary across studies: some classify the phenotype according to body fat percentage, whereas others additionally require metabolic abnormalities; therefore, normal-weight obesity and metabolically abnormal normal weight should not be treated as synonymous conditions [10,12]. Differences in cutoff points, measurement methods, sex, age, and population characteristics account for part of the variation observed in prevalence estimates; for example, a study using the same sex-specific InBody-based thresholds as the present study, conducted among Slovak university-aged adults, reported a normal-weight obesity prevalence of 19.0% in men and 31.5% in women, underscoring how population and methodological factors shape these estimates [13].

In the present study, the term hidden adiposity is used operationally to describe the coexistence of a normal BMI and a body fat percentage above the sex-specific operational threshold used in this study. The population-specific validity of these body-fat thresholds has not been established for Ecuadorian university students; therefore, they are treated as operational research criteria rather than diagnostic thresholds.

The term ‘hidden’ does not imply that the condition is visually imperceptible or that metabolic abnormalities are present; rather, it indicates that elevated body fat is not detected by conventional BMI-based classification. This distinction prevents a research category from being interpreted as a clinical diagnosis and makes it possible to examine whether BMI-defined normal weight status encompasses heterogeneous body-composition and behavioral profiles.

Bioelectrical impedance analysis represents a feasible complementary approach for expanding body-composition assessment. The technique estimates body composition from the electrical properties of body tissues and provides indicators such as body fat percentage, fat mass, fat-free mass, and skeletal muscle mass [14]. Its speed, non-invasive nature, and applicability to large samples explain its widespread use in epidemiological studies and non-hospital settings. Nevertheless, the validity of its estimates depends on the type of device, the predictive equations employed, and the degree of procedural standardization. Factors such as hydration status, recent food intake, prior physical activity, and certain body characteristics may introduce variability into the results [14,15,16]. Accordingly, bioelectrical impedance analysis is not equivalent to reference methods such as multicomponent models, magnetic resonance imaging, or dual-energy X-ray absorptiometry. Its principal value in this context lies in providing body-composition estimates that are not available from body weight or BMI alone, provided that measurement conditions are adequately controlled.

Because this study combines device-derived body-composition estimates with self-reported lifestyle data, it also provides a limited context for considering datafication in university health. Here, datafication is used narrowly to refer to the transformation and joint analysis of bodily and behavioral information as structured data [17,18]. The study did not examine continuous digital monitoring, administrative data linkage, institutional surveillance, algorithms, or automated decision-making; accordingly, datafication is considered a secondary interpretive framework rather than an empirical outcome of the study.

The literature on university health has addressed physical activity, nutrition, sleep, stress, and well-being. In Latin America, evidence from multidimensional health assessments in university populations remains limited. Recent research in a Colombian Andean university community incorporated anthropometric, hemodynamic, and biochemical indicators while highlighting the need for multidimensional approaches to university health [19]. Regional research involving health sciences students in Peru has also examined BMI-defined overweight and obesity in relation to physical activity, diet, and other lifestyle factors [20]. However, the joint examination of body composition, hidden adiposity, and multidimensional lifestyle profiles remains relatively underexplored in Latin American university populations, particularly in Andean settings. This gap limits understanding of whether associations reported in other populations are reproduced within the social, cultural, and educational contexts of the region.

Against this background, the objective of the present study was to examine BMI–body-fat classification discordance and compare body-composition and lifestyle profiles across operational adiposity phenotypes among sport sciences students at Universidad Técnica del Norte, Ecuador. Specifically, BMI classification performance relative to the operational BIA-based body-fat criterion was assessed, body-composition indicators and HPLP-II domains were compared across phenotypes, and factors associated with hidden adiposity were explored within the normal-BMI subgroup.

Based on this rationale, it was hypothesized that BMI would show limited sensitivity for identifying elevated body fat relative to the operational BIA-based criterion. Among participants with normal BMI, differences in body-composition indicators and HPLP-II domains between those with and without hidden adiposity were examined as secondary analyses. Associations of hidden adiposity with sex, degree program, age, and the overall 52-item HPLP-II mean score, as well as the identification of lifestyle profiles, were examined as exploratory analyses. Owing to the cross-sectional design, all identified relationships were interpreted as associations rather than causal effects.

## 2. Materials and Methods

### 2.1. Study Design and Setting

An analytical cross-sectional observational study was conducted in May 2026 at Universidad Técnica del Norte in Ibarra, Ecuador. The source population comprised students enrolled in the Physical Activity and Sport Pedagogy and Sports Training degree programs.

The study integrated two sources of information obtained directly from the participants: body-composition measurements performed using bioelectrical impedance analysis and responses to the Health-Promoting Lifestyle Profile II (HPLP-II). Academic records, medical records, administrative records, and other institutional databases were not used. The description of the study design, participant selection, variables, and analytical procedures was structured in accordance with the STROBE guidelines for observational studies [21].

### 2.2. Participants and Sampling

A non-probability convenience sampling strategy was used. Recruitment targeted students from the two participating degree programs and was conducted after authorization had been obtained from their respective program coordinators.

The inclusion criteria were as follows: being 18 years of age or older; being enrolled in one of the degree programs included in the study; voluntarily agreeing to participate through electronic informed consent; completing the HPLP-II questionnaire; having a valid InBody 270 measurement; and providing a research code that enabled linkage between the two data sources.

Responses without informed consent, incomplete questionnaires, technically invalid InBody 270 measurements, records that could not be unambiguously linked, and duplicate records that could not be resolved using the established data-cleaning rules were excluded. Technically invalid InBody 270 measurements were defined as assessments that did not produce a complete device-generated result or were flagged as invalid by the analyzer. Missing data were not imputed.

Initially, 392 records from the InBody 270 and 376 questionnaire responses were available. After verifying data completeness, resolving duplicate records, and linking both instruments through the code assigned to each participant, the final analytical sample comprised 303 students with complete data.

Because recruitment was voluntary and non-probabilistic and was restricted to two sport sciences degree programs, the findings describe the study sample and should not be interpreted as representative of all students at Universidad Técnica del Norte, students from other academic fields, or the broader Ecuadorian university population.

No a priori sample size calculation was performed. Recruitment was based on voluntary participation during the study period, and all eligible students who volunteered and provided complete linked data were included in the analytical sample.

### 2.3. Recruitment and Data Collection Procedure

Before data collection, a formal request for authorization was submitted to the coordinators of the Physical Activity and Sport Pedagogy and Sports Training degree programs. The request included the data-collection schedule and a description of the planned procedures. After authorization was obtained from the academic coordinators, the research team presented the study directly to students in their classrooms. During these sessions, the study purpose, planned activities, voluntary nature of participation, and confidential handling of information were explained. Interested students accessed a digital form, reviewed the informed consent information, and explicitly recorded their decision to participate. Only students who provided consent continued in the study.

Participants then completed the Health-Promoting Lifestyle Profile II (HPLP-II) and underwent body-composition assessment. The two procedures were linked using a unique alphanumeric code assigned to each participant to protect confidentiality and ensure data integrity. The sequence comprised electronic informed consent, questionnaire completion, InBody 270 assessment, verification of record consistency, and construction of the analytical dataset.

### 2.4. Institutional Review, Ethical Safeguards, and Informed Consent

The research protocol was reviewed and approved by the Research Ethics Committee of the Faculty of Education, Science, and Technology at Universidad Técnica del Norte, under Approval Certificate No. UTN-FECYT-CEI-2024-006-C, dated 10 October 2025. The study was conducted in accordance with the principles of the Declaration of Helsinki, applicable national regulations, and institutional provisions governing research involving human participants. The project, identified as Project No. 0000001437, also received scientific and administrative approval from the Research Council of Universidad Técnica del Norte through Resolution No. UTN-CI-2025-250-R.

The study included adult participants and involved non-invasive procedures. It did not include clinical interventions, biological sample collection, or any modification of students’ behaviors. Measures related to autonomy, voluntariness, risk minimization, privacy, and confidentiality were implemented in accordance with the ethical principles governing research involving human participants [22].

Before the study procedures began, each participant received information regarding the study objectives, planned activities, scientific use of the data, potential risks and benefits, voluntary nature of participation, confidentiality of the information, and the right to withdraw at any time without academic or administrative consequences. Informed consent was recorded electronically through the digital form. Only students who explicitly selected the consent option proceeded to complete the HPLP-II and undergo body-composition assessment using the InBody 270. Declined responses were not included in the analytical dataset.

### 2.5. Instruments and Measurements

#### 2.5.1. Body Composition Assessment

Body composition was estimated using an InBody 270 analyzer (InBody Co., Ltd., Seoul, Republic of Korea), which employs multifrequency segmental bioelectrical impedance analysis. Bioelectrical impedance analysis estimates body components based on the opposition of body tissues to the passage of low-intensity electrical currents. Although this is a rapid and non-invasive procedure, its results should be interpreted as estimates that depend on the device and measurement conditions rather than as equivalents to multicomponent methods or dual-energy X-ray absorptiometry [14,15,16].

The device was operated using its default adult configuration. Manufacturer guidance recommends standing upright for approximately 5 min before testing, avoiding food intake before the assessment or postponing testing for at least 2 h after a meal, using the bathroom beforehand, avoiding exercise before testing, and performing the assessment in the morning when possible [23].

Standing height was measured before bioelectrical impedance assessment using a stadiometer, with participants barefoot and in an upright position. The measured height was used for the calculation of BMI and height-adjusted body-composition indices.

Before measurement, participants removed their shoes, socks, and heavy accessories. The soles of their feet and their hands were checked to ensure that they were free of lotions that could interfere with electrical contact. Participants remained standing for approximately 5 min before stepping onto the device and were assessed in an upright position, with their feet aligned on the foot electrodes, their thumbs placed on the hand electrodes, and their arms extended and held away from the trunk. The preparation procedures standardized in the present study included removal of shoes, socks, and heavy accessories; lotion-free palms and soles; a premeasurement standing period; standardized electrode contact and body position; and remaining still throughout the assessment. Recent food intake, previous exercise, hydration status, bladder emptying, and time of day were not individually verified or standardized.

The following body-composition variables were obtained from the device-generated report:

- Body weight (kg);
- Body fat mass (kg);
- Body fat percentage;
- Fat-free mass (kg);
- Skeletal muscle mass (kg);
- Skeletal muscle index;
- Fat-free mass index;
- Fat mass index;
- Visceral fat level.

BMI was calculated from body weight and stadiometer-measured height as weight in kilograms divided by height in meters squared. Fat mass index and fat-free mass index represent the corresponding body components adjusted for height squared. Skeletal muscle index and visceral fat level were obtained directly from the device-generated report.

#### 2.5.2. Health-Promoting Lifestyle Profile II

Health-promoting behaviors were assessed using a 52-item Spanish-language adaptation of the Health-Promoting Lifestyle Profile II (HPLP-II). The adapted form used in the present study corresponds to the initial 52-item version reported during the adaptation process conducted by Balbé et al. in Mexican university students [6], rather than to the subsequent reduced 16-item, four-factor HPLP-II UM solution identified by those authors. The 52-item form was retained to provide a broad assessment of the six adapted behavioral domains and to permit domain-level analyses consistent with the multidimensional framework of the present study. Because this 52-item adapted structure was not the final factorial solution reported by Balbé et al., factorial equivalence was not assumed for the present Ecuadorian sample.

The 52 items were distributed across six domains: interpersonal relations (9 items), nutrition (10 items), health responsibility (7 items), physical activity (9 items), stress management (8 items), and positive psychology (9 items). In the original HPLP-II, the corresponding sixth domain is designated as spiritual growth [5]. In the Mexican adaptation used here, this domain was relabeled positive psychology [6], and this terminology was therefore retained throughout the analyses and presentation of results.

Each item was rated on a four-point Likert scale: never (1), sometimes (2), often (3), and routinely (4). The overall 52-item mean score and domain-specific mean scores were calculated, with higher values indicating a greater reported frequency of health-promoting behaviors. The overall mean was treated as an observed composite summary score of the adapted 52-item form and was not interpreted as evidence of a unidimensional latent construct.

Internal consistency was examined using Cronbach’s alpha for the overall 52-item composite and each of the six domain scores. Cronbach’s alpha was interpreted solely as an index of internal consistency and not as evidence of construct validity or factorial equivalence. To our knowledge, local factorial validation of this adapted 52-item structure has not been established in Ecuadorian university students.

No confirmatory factor analysis was conducted in the present study; therefore, the six-domain structure was used as an adapted analytical framework and was not interpreted as locally validated.

### 2.6. Operational Definitions and Body Phenotypes

BMI classification was based on the criteria commonly applied to adult populations. Underweight was defined as a BMI below 18.5 kg/m^2^; normal BMI as 18.5–24.9 kg/m^2^; overweight as 25.0–29.9 kg/m^2^; and BMI-defined obesity as a BMI of 30.0 kg/m^2^ or higher. For the classification analysis, all values of 25.0 kg/m^2^ or higher were grouped as elevated BMI [24].

Body fat percentage was interpreted according to the sex-specific operational thresholds configured in the InBody 270 report and its technical documentation. Elevated body fat was defined a priori as >20% in men and >28% in women. Comparable sex-specific thresholds (>20% in men and >28% in women) have been used in recent research involving young adults assessed using InBody devices, including a study of Slovak university-aged adults that adopted the same manufacturer-based cutoffs [13]. However, that evidence derives from a Central European population and does not establish the validity of these thresholds for Latin American or, specifically, Ecuadorian university students. These thresholds were specified before outcome classification and were not derived from the distribution of body-fat values in the current sample. The same sex-specific thresholds were applied identically to every participant through the InBody 270 built-in classification algorithm, with no manual adjustment or participant-specific modification by the research team. Their population-specific validity has not been established for Ecuadorian university students; consequently, they were treated as operational research criteria rather than diagnostic thresholds.

This distinction is important because BMI is an indirect indicator and may fail to identify all individuals with elevated adiposity [8]. Likewise, an elevated body fat percentage alone does not establish clinical obesity, which requires evidence of organ dysfunction and/or meaningful functional limitations attributable to excess adiposity [9].

Five body phenotypes were defined:

- Underweight: BMI < 18.5 kg/m^2^.
- Normal BMI and normal body fat: BMI 18.5–24.9 kg/m^2^ and BF ≤ 20% in men or ≤28% in women.
- Hidden adiposity: BMI 18.5–24.9 kg/m^2^ and BF > 20% in men or >28% in women.
- Elevated BMI and normal body fat: BMI ≥ 25.0 kg/m^2^ and BF ≤ 20% in men or ≤28% in women.
- Elevated BMI and elevated body fat: BMI ≥ 25.0 kg/m^2^ and BF > 20% in men or >28% in women.

For the dichotomous BMI classification-performance analysis, BMI < 25.0 kg/m^2^ was treated as non-elevated BMI and therefore included both underweight and normal-BMI participants; phenotype-specific analyses retained the underweight group as a separate category.

Hidden adiposity was the primary outcome. The term was used to identify a discordance between BMI classification and estimated body composition, without implying a clinical diagnosis.

### 2.7. Data Management and Quality Control

The datasets generated by the InBody 270 and the HPLP-II were reviewed separately before integration. Quality-control procedures included identifying missing codes, incomplete responses, values outside the possible ranges, inconsistencies among basic variables, and duplicate records.

Each student was identified using an alphanumeric code generated specifically for the study. This code was used exclusively to link the questionnaire data with the body-composition measurement. Names, personal identification numbers, email addresses, telephone numbers, and additional academic information were not included in the analytical dataset.

When more than one valid InBody measurement was associated with the same code, the most recent measurement was retained. For duplicate questionnaires, the first complete and valid response was kept. Records that could not be unequivocally matched across the two instruments were excluded.

The final dataset included only the 303 complete cases. Missing questionnaire responses or body-composition measurements were not imputed. The data were stored in a password-protected file, and access was restricted to the research team (Table 1).

### 2.8. Statistical Analysis

Analyses were performed using IBM SPSS Statistics for Windows, version 25.0 (IBM Corp., Armonk, NY, USA) [25]. All statistical tests were two-tailed, and the significance level was set at *p* < 0.05.

Categorical variables were summarized using frequencies and percentages. For continuous variables, the mean, standard deviation, median, 25th and 75th percentiles, minimum, and maximum were calculated. The internal consistency of the adapted 52-item HPLP-II was estimated using Cronbach’s alpha for the overall 52-item composite and each of the six domain scores.

Because the sample was markedly imbalanced by sex (78.9% men, 21.1% women), prevalence estimates and the principal body-composition indicators were additionally examined by sex wherever subgroup sizes permitted stable estimation, and odds ratios involving sex are reported with corresponding confidence intervals to convey the precision of these estimates. A formal test of the interaction between sex and degree program was not conducted because the resulting cross-classified subgroups were considered too small to yield a stable estimate; this constitutes a limitation of the present analysis.

The distribution of continuous variables was assessed using the Kolmogorov–Smirnov and Shapiro–Wilk tests. Because most body-composition and behavioral indicators were not normally distributed, non-parametric procedures were prioritized.

The association between elevated BMI and elevated body fat was evaluated using Pearson’s chi-square test. The strength of the association was expressed using the phi coefficient and Cramer’s V. Agreement between the two classification criteria was estimated using Cohen’s kappa coefficient [26].

Using BIA-estimated elevated body fat as the operational comparison criterion, sensitivity, specificity, positive predictive value, negative predictive value, false-negative rate, and overall accuracy were calculated for the BMI cutoff of ≥25.0 kg/m^2^. These indicators were interpreted as measures of classification performance within the study sample rather than as validation against a clinical diagnostic reference standard.

The normal-BMI and normal-body-fat group was compared with the hidden-adiposity group using the Mann–Whitney U test. Effect size was calculated using the following expression:

$$r=\frac{\left|Z\right|}{\sqrt{N}}$$

where |*Z*| denotes the standardized test statistic, and *N* represents the number of participants included in the comparison.

Differences among the four principal body phenotypes were analyzed using the Kruskal–Wallis H test. The five participants classified as underweight were excluded from this comparison because of the small size of that group. When the overall test was statistically significant, pairwise comparisons were conducted using the Bonferroni correction. The overall effect size was expressed as epsilon-squared.

Associations between the overall 52-item HPLP-II mean score, the six domain-specific mean scores, and body-composition indicators were estimated using Spearman’s rho. Because multiple related comparisons were conducted, Bonferroni adjustment was applied to post hoc pairwise comparisons following significant Kruskal–Wallis tests. No global family-wise correction was applied across all secondary and exploratory analyses; consequently, these findings were interpreted with emphasis on effect sizes, confidence intervals where applicable, consistency across analyses, and their exploratory nature rather than on statistical significance alone.

A binary logistic regression model was constructed for the subgroup of participants with a normal BMI. The dependent variable was the presence of hidden adiposity, coded as 1, compared with the normal-BMI and normal-body-fat phenotype, coded as 0. Sex, degree program, age, and the overall 52-item HPLP-II mean score were included as explanatory variables.

These variables were selected to provide a parsimonious exploratory model representing demographic characteristics (sex and age), academic context (degree program), and overall health-promoting lifestyle (overall 52-item HPLP-II mean score). The overall 52-item HPLP-II mean score was entered instead of the six domain-specific mean scores to limit model dimensionality and avoid redundant lifestyle predictors.

Linearity in the logit for the continuous predictors (age and the overall 52-item HPLP-II mean score) was assessed using the Box–Tidwell procedure; the interaction terms between each continuous predictor and its natural logarithm were not statistically significant (*p* > 0.10 for both), indicating no evidence of departure from linearity. Multicollinearity among predictors was examined using variance inflation factors (VIFs), which ranged from 1.02 to 1.18, well below common thresholds of concern (VIF < 5), indicating that multicollinearity did not materially affect the stability of the coefficient estimates.

Results are expressed as regression coefficients, standard errors, odds ratios, 95% confidence intervals, and *p* values. Model fit was assessed using the omnibus test, the Cox and Snell and Nagelkerke coefficients, and the classification table. Model calibration was evaluated using the Hosmer–Lemeshow test [27].

Finally, an exploratory cluster analysis was conducted using the k-means procedure [28]. A three-cluster solution was selected a priori to represent less favorable, intermediate, and more favorable lifestyle profiles while maintaining adequate cluster sizes. The analysis incorporated the six HPLP-II domains (physical activity, nutrition, health responsibility, stress management, interpersonal relations, and positive psychology); the overall 52-item HPLP-II mean score was excluded from the clustering variables because it is mathematically derived from these six domains, and its inclusion would have introduced redundancy into the distance-based clustering procedure. Because the six domains are expressed on the same 1–4 response scale, the clustering variables were standardized (z-scores) before the k-means procedure to prevent domains with slightly larger variances from disproportionately influencing the Euclidean-distance-based solution. The default iteration and convergence parameters in SPSS 25 were used.

Because the overall HPLP-II score is not used to define the clusters and cluster stability was not formally assessed (e.g., through bootstrap resampling or split-sample replication), the clusters were interpreted as an exploratory segmentation rather than as independent clinical categories. The ANOVA table automatically generated by SPSS was used only to describe the relative contribution of each variable to cluster separation. The association between cluster membership and body phenotypes was examined using Pearson’s chi-square test and Cramer’s V.

## 3. Results

### 3.1. Sample Characteristics and Distribution of Body Phenotypes

The analytical sample comprised 303 students, with a predominance of male participants. Representation from the Physical Activity and Sport Pedagogy and Sports Training degree programs was relatively balanced. Although 60.7% of participants had a body mass index within the normal range, 53.5% had an elevated body fat percentage according to the sex-specific criteria established for the study.

Hidden adiposity was identified in 19.5% of the total sample. Among the 184 students with a normal BMI, 59 had elevated body fat, corresponding to 32.1% of this subgroup. Table 2 presents the sociodemographic characteristics, principal body-composition indicators, and distribution of body phenotypes.

### 3.2. Health-Promoting Lifestyle Profile II Scores and Internal Consistency

The overall 52-item HPLP-II mean score was 2.53. Among the domains assessed, positive psychology had the highest mean score, followed by interpersonal relations. Health responsibility had the lowest mean score, whereas physical activity, stress management, and nutrition showed intermediate values.

The overall 52-item composite yielded a Cronbach’s alpha of 0.941, while domain-specific coefficients ranged from 0.741 to 0.854. Physical activity and positive psychology showed the highest coefficients. Descriptive and reliability results are presented in Table 3.

### 3.3. Agreement Between BMI Classification and Body Fat Classification

BMI classification was significantly associated with the presence of elevated body fat, χ^2^(1) = 99.94, *p* < 0.001. The magnitude of this association was moderate to high, with both the phi coefficient and Cramer’s V equal to 0.574. Nevertheless, agreement between the two criteria was incomplete, with a Cohen’s kappa coefficient of 0.546.

Of the 162 students with elevated body fat, 103 were identified using a BMI cutoff of ≥25.0 kg/m^2^, whereas 59 had a non-elevated BMI. These 59 cases corresponded to the hidden-adiposity phenotype. Conversely, 11 participants had an elevated BMI despite having a body fat percentage within the operational normal range.

Using elevated body fat estimated by the InBody 270 as an operational classification criterion, BMI showed 63.6% agreement for identifying elevated body fat and 92.2% agreement for identifying normal body fat. The corresponding positive and negative classification agreement values were 90.4% and 68.8%, respectively, and the overall percentage of concordant classifications was 76.9%. The false-negative rate was 36.4%, indicating that BMI failed to identify more than one-third of participants classified as having elevated body fat by bioelectrical impedance analysis. Because bioelectrical impedance analysis is not equivalent to a criterion method such as dual-energy X-ray absorptiometry or a multicomponent model, these indicators are interpreted as measures of classification agreement between BMI and an operational BIA-based criterion, rather than as diagnostic accuracy in the clinical sense. The results are presented in Table 4.

### 3.4. Comparison Between Students with Normal BMI and Normal Body Fat and Those with Hidden Adiposity

The analysis was restricted to the 184 participants whose BMI fell within the normal range. Of these, 125 had a normal body fat percentage, whereas 59 met the operational criterion for hidden adiposity.

The overall 52-item HPLP-II mean score was lower in the hidden-adiposity group; however, the difference did not reach statistical significance (U = 4283.5, *p* = 0.077). No statistically significant differences were observed in nutrition, interpersonal relations, or positive psychology.

Students with hidden adiposity had significantly lower scores for physical activity (*p* < 0.001), health responsibility (*p* = 0.030), and stress management (*p* = 0.031). Among the behavioral domains, physical activity showed the largest difference (r = 0.253).

The most pronounced differences were observed in the body-composition indicators. The hidden-adiposity group had lower skeletal muscle mass, a lower fat-free mass index, and a lower skeletal muscle index. In contrast, this group showed higher values for fat mass index, visceral fat level, and body fat percentage, with particularly large effect sizes for visceral fat level (r = 0.774), fat mass index (r = 0.765), and body fat percentage (r = 0.757). Because body fat percentage was one of the criteria used to define hidden adiposity, and fat mass index and visceral fat level are closely related adiposity indicators, these differences partly reflect the operational definition of the phenotype rather than constituting fully independent findings. In contrast, the differences observed in skeletal muscle mass, fat-free mass index, skeletal muscle index, and physical activity were not used to construct the phenotype and therefore represent independent characteristics associated with hidden adiposity. The complete results are presented in Table 5.

### 3.5. Differences Among Body Phenotypes

The comparison included 298 participants distributed across four body phenotypes: normal BMI and normal body fat, hidden adiposity, elevated BMI with normal body fat, and apparent adiposity. The five participants classified as underweight were excluded because of the small size of this group.

Significant differences among the phenotypes were observed for the overall 52-item HPLP-II mean score (H = 11.33, *p* = 0.010), physical activity (H = 22.82, *p* < 0.001), health responsibility (H = 12.63, *p* = 0.006), and stress management (H = 8.10, *p* = 0.044). No significant differences were found for nutrition, interpersonal relations, or positive psychology. Effect sizes for the behavioral variables were small, with physical activity showing the largest magnitude (ε^2^ = 0.067).

The most pronounced differences were observed in body-composition indicators. Visceral fat level showed the largest effect size (ε^2^ = 0.758), followed by fat mass index (ε^2^ = 0.747) and body fat percentage (ε^2^ = 0.674). As in the two-group comparison described above, these large effect sizes partly reflect the fact that body fat percentage, fat mass index, and visceral fat level are directly or closely related to the criteria used to define the body phenotypes, rather than constituting fully independent evidence of phenotypic differentiation. Relevant differences were also observed in fat-free mass index, skeletal muscle index, and skeletal muscle mass, which were not used to construct the phenotypes and can therefore be interpreted as independent findings.

Bonferroni-adjusted pairwise comparisons showed that the hidden-adiposity group had lower physical activity scores than the normal-BMI and normal-body-fat group. The two groups also differed significantly in skeletal muscle mass, fat-free mass index, skeletal muscle index, fat mass index, visceral fat level, and body fat percentage. The overall results are presented in Table 6.

### 3.6. Associations Between Health-Promoting Lifestyles and Body Composition

HPLP-II scores were significantly associated with several body-composition indicators; however, all correlation coefficients were small in magnitude, with absolute values ranging from 0.120 to 0.229.

The overall 52-item HPLP-II mean score was positively correlated with skeletal muscle mass, skeletal muscle index, and fat-free mass index. It was also inversely correlated with body fat percentage. Among the specific domains, physical activity showed the most consistent pattern: it was positively associated with all three muscle-related indicators and negatively associated with body fat percentage and fat mass index.

Health responsibility and stress management were also positively associated with muscle-related indicators and inversely associated with body fat percentage. Nutrition and positive psychology were associated only with variables related to the muscular component. No statistically significant correlations were observed for interpersonal relations.

Taken together, these associations suggest that a higher frequency of health-promoting behaviors coexisted with slightly more favorable muscle-related indicators and lower adiposity. Nevertheless, the small magnitude of the coefficients indicates that lifestyle scores accounted for only a limited proportion of the variability in body composition. The results are presented in Table 7.

### 3.7. Factors Associated with Hidden Adiposity Among Students with Normal BMI

The logistic regression analysis included 184 participants with a normal BMI. Of these, 59 (32.1%) had hidden adiposity, whereas 125 (67.9%) had both a normal BMI and normal body fat.

The model was statistically significant, χ^2^(4) = 32.39, *p* < 0.001, and explained 22.6% of the variance in the outcome according to Nagelkerke’s R^2^. The Hosmer–Lemeshow test was not statistically significant, χ^2^(8) = 5.93, *p* = 0.655, indicating no evidence of poor model fit.

Female sex was associated with higher odds of hidden adiposity. Women had 5.13 times the odds observed in men (OR = 5.13; 95% CI: 2.23–11.83; *p* < 0.001). In contrast, enrollment in the Sports Training degree program was associated with lower odds of the outcome compared with enrollment in the Physical Activity and Sport Pedagogy degree program (OR = 0.28; 95% CI: 0.14–0.59; *p* < 0.001).

Age and the overall 52-item HPLP-II mean score were not independently associated with hidden adiposity. Although the model correctly classified 73.4% of participants, its sensitivity for identifying hidden adiposity was only 27.1%, whereas specificity reached 95.2%. Therefore, the model identified participants without hidden adiposity considerably more accurately than those with this phenotype, and the overall classification accuracy should not be interpreted as evidence of adequate predictive performance. Given this pattern, the model is best regarded as explanatory/exploratory rather than predictive, and it should not be used as an individual screening tool. The results are presented in Table 8.

### 3.8. Lifestyle Profiles and Their Relationship with Body Phenotypes

Exploratory k-means cluster analysis, performed using the six HPLP-II domains (physical activity, nutrition, health responsibility, stress management, interpersonal relations, and positive psychology), identified three distinct health-promoting lifestyle profiles. The overall 52-item HPLP-II score was excluded from the clustering variables because it is mathematically derived from these six domains, and its inclusion would have introduced redundancy into the distance-based clustering procedure. Profile 1 comprised 118 participants and showed the lowest scores across all six domains. Profile 2 included 131 students and showed intermediate values, whereas Profile 3 comprised 54 participants and had the highest scores.

The overall 52-item HPLP-II mean score, although not itself used to define the clusters, increased progressively across profiles as a consequence of its constituent domains, from 2.18 in Profile 1 to 2.58 in Profile 2 and 3.09 in Profile 3. Accordingly, the clusters were labeled the less favorable profile, intermediate profile, and more favorable profile, respectively.

To examine the relationship between lifestyle profiles and body phenotypes, the five participants classified as underweight were excluded. The distribution of body phenotypes differed significantly across the three profiles, χ^2^(6) = 14.87, *p* = 0.021. However, the magnitude of the association was small, with a Cramer’s V of 0.158.

Hidden adiposity was present in 26 participants in the less favorable profile, 26 in the intermediate profile, and 7 in the more favorable profile. Although its relative frequency decreased as the lifestyle profile improved, the weak association indicates that behavioral scores alone were insufficient to determine body phenotype. The results are presented in Table 9.

## 4. Discussion

### 4.1. General Interpretation of the Findings

The central finding was the discordance between conventional classification based on body mass index and body composition estimated by bioelectrical impedance analysis. Although 60.7% of the students had a BMI within the normal range, 53.5% of the sample had an elevated body fat percentage. Hidden adiposity was also present in 19.5% of the total sample and in 32.1% of participants with a normal BMI. In practical terms, approximately one in three students classified as normal according to BMI had an elevated body fat level based on the cutoff points used.

These results support the primary hypothesis of the study: BMI, when used in isolation, does not adequately represent the heterogeneity of body composition among university students. The indicator showed high specificity but a sensitivity of only 63.6%, with a false-negative rate of 36.4%. Therefore, it was more effective in confirming the presence of adiposity among individuals with an elevated BMI than in ruling out excess body fat among those within the normal BMI range.

This pattern is consistent with the accumulated evidence questioning the use of BMI as a direct proxy for adiposity. BMI does not distinguish between fat mass, muscle mass, body water, or the regional distribution of adipose tissue; therefore, individuals with the same weight-to-height relationship may have substantially different body compositions [8,11,15]. Recent international discussions have also proposed distinguishing the presence of excess adiposity from the diagnosis of clinical obesity, which requires evidence of organ dysfunction, functional limitation, or metabolic impairment attributable to adipose tissue [9].

The secondary hypothesis—that hidden adiposity would be associated with less favorable lifestyle profiles—received partial support. Students with this phenotype had lower scores for physical activity, health responsibility, and stress management. However, significant differences were not observed across all HPLP-II domains, and the overall score did not reach statistical significance in the specific comparison between the two normal-BMI groups. Behavioral associations were present, but they were selective and small in magnitude.

### 4.2. BMI, Hidden Adiposity, and Body-Composition Profiles

The limited sensitivity of BMI observed in this study is consistent with the meta-analysis by Okorodudu et al., which documented that BMI may misclassify a relevant proportion of individuals with elevated adiposity [8]. Similarly, Gómez-Ambrosi et al. showed that traditional BMI classification fails to identify individuals with excess body fat and cardiometabolic risk factors [11]. The problem is not that BMI is entirely devoid of utility, but rather that its performance is insufficient for interpreting individual body composition.

The identification of hidden adiposity should not automatically be equated with a diagnosis of clinical obesity. The present study did not assess blood pressure, lipid profile, glucose, insulin resistance, liver function, systemic inflammation, or organ damage. Therefore, the term hidden adiposity is more precise than “hidden obesity.” This concept describes the coexistence of a normal BMI and an elevated body fat percentage without attributing a disease that was not evaluated. The term apparent adiposity, used for the phenotype combining elevated BMI with elevated body fat, was retained for terminological symmetry with hidden adiposity rather than as an established clinical category, and it likewise does not imply confirmed clinical obesity or metabolic disease.

Terminological caution does not diminish the importance of the finding. Previous studies have linked elevated adiposity among individuals with normal weight to cardiometabolic abnormalities and increased cardiovascular risk [9,11]. However, this evidence does not imply that all participants identified in the present study had the same abnormalities. The results should be interpreted as evidence of morphological discordance warranting further assessment, rather than as confirmation of disease.

Students with hidden adiposity not only had a higher body fat percentage but also showed a higher fat mass index and visceral fat level, together with lower skeletal muscle mass, fat-free mass index, and skeletal muscle index. This combination indicates that the phenotype was characterized by a different relationship between adipose and muscular components. Body-composition assessment therefore provided information that weight and height alone could not offer [12,13].

The largest effect sizes were observed for visceral fat level, fat mass index, and body fat percentage. These findings were partly expected because these variables contributed directly or indirectly to the definition of the phenotypes. Accordingly, they should not be interpreted as independent associations, but rather as evidence that the operational classification produced groups with distinct body-composition profiles. The differences in muscular indicators are more informative, although they also require caution because of the sex composition of the groups.

### 4.3. Differences According to Sex and Degree Program

Female sex was associated with higher odds of hidden adiposity in the multivariable model. Women had approximately five times the odds observed among men. This result should be interpreted in light of the predominantly male composition of the sample and the use of sex-specific body fat cutoff points.

Sex differences in the distribution of fat mass and fat-free mass may influence both adiposity classification and the values of muscle-related indicators. Therefore, the result should not be interpreted as evidence that female sex independently causes hidden adiposity. The association may reflect biological differences, classification criteria, unequal sample composition, and behavioral or sociocultural factors not included in the model.

Enrollment in the Sports Training degree program was associated with lower odds of hidden adiposity compared with enrollment in the Physical Activity and Sport Pedagogy degree program. Sports Training students may have greater exposure to systematic sports practice, resistance training, exercise planning, or prior selection processes related to their sporting backgrounds. However, none of these variables was measured directly.

Consequently, the degree program cannot be said to exert a protective effect. The association may also reflect self-selection: students with greater sporting experience or a particular body composition may be more likely to choose Sports Training. Future analyses should incorporate training history, weekly frequency, intensity, sporting discipline, years of practice, and competitive participation.

Age was not a significant predictor, probably because the sample was concentrated within a relatively narrow age range. University life represents a transitional period during which patterns of eating, movement, rest, and self-care become established and may persist into adulthood [29]. Nevertheless, the limited variability in age reduced the ability to detect differences associated with this variable.

### 4.4. Physical Activity, Health Responsibility, and Stress Management

Physical activity was the behavioral dimension showing the most consistent relationship with body-composition indicators. Students with hidden adiposity had lower scores, and in the correlational analysis, higher physical activity scores were associated with greater skeletal muscle mass, skeletal muscle index, and fat-free mass index. Physical activity was also inversely associated with body fat percentage and fat mass index.

The direction of these associations is consistent with the role of exercise in preserving and developing muscle tissue. However, the coefficients were small, and the cross-sectional design does not allow the conclusion that higher physical activity scores produced a more favorable body composition. The HPLP-II measures the perceived frequency of behaviors and does not provide a detailed quantification of intensity, duration, energy expenditure, training volume, or sedentary behavior.

The literature shows that insufficient physical activity and excess weight are common among university students across different cultural contexts [30,31]. Sedentary behavior and physical activity are also associated with perceived health, well-being, and life satisfaction, although these relationships depend on personal and contextual factors.

Lower scores for health responsibility may reflect a lower frequency of self-care behaviors, information seeking, preventive monitoring, or conscious use of healthcare resources. Nevertheless, this finding should not be used to assign individual responsibility to students for their body composition. University lifestyles are shaped by schedules, academic workload, financial resources, food availability, transportation, living arrangements, social networks, and institutional opportunities.

Stress management scores were also lower in the hidden-adiposity group. The relationship between stress and body composition may be mediated by changes in sleep, eating behavior, sedentary behavior, and emotional regulation. However, the effect size was small, and no stress biomarkers were assessed. University-based stress-management programs may improve psychological outcomes, but their effectiveness depends on the nature of the intervention and the conditions of the academic environment.

The transition to higher education may simultaneously alter body weight, physical activity, sedentary behavior, and eating patterns. For this reason, body composition and health should not be interpreted solely as the result of individual choices. The university functions as an environment that structures opportunities for movement, meal schedules, rest periods, and exposure to stressful situations.

### 4.5. Absence of Generalized Differences in the HPLP-II

No significant differences between the normal-BMI groups were observed for nutrition, interpersonal relations, or positive psychology. The overall 52-item HPLP-II mean score was also not independently associated with hidden adiposity. These results indicate that the phenotype was not accompanied by a uniform deterioration across all lifestyle components.

The absence of an association with the overall score may be explained by the fact that the average of the 52 responses combines heterogeneous domains. A student may have a high score for interpersonal relations or positive psychology while simultaneously reporting a low physical activity score. When all components are averaged, specific differences may be attenuated.

This finding illustrates the analytical value of examining domain-specific scores rather than relying exclusively on the overall 52-item composite. A behavioral scale cannot replace a body-composition measurement, and a body-composition measurement alone cannot be used to infer the overall quality of an individual’s lifestyle.

The observed Cronbach’s alpha coefficients indicate internal consistency of the composite and domain scores in this sample, but they do not establish the factorial validity of the six-domain, 52-item adapted structure in Ecuadorian students. Reliability is only one psychometric property. In addition, cultural and linguistic equivalence between the Mexican adaptation and the present Ecuadorian university context was not independently evaluated, which may have influenced the interpretation or functioning of individual items. Further evaluation of factorial structure, convergent and discriminant validity, and measurement invariance by sex and degree program would be needed to strengthen evidence for the validity of this adapted form in the local context.

### 4.6. Lifestyle Profiles and Student Heterogeneity

Cluster analysis identified three profiles ordered from the lowest to the highest frequency of health-promoting behaviors. The existence of these profiles confirms that the sample was not behaviorally homogeneous. Some students had consistently low scores, others showed intermediate values, and a third group had high scores across all domains.

The identification of these profiles is consistent with research demonstrating the coexistence of distinct clusters of healthy and risk-related behaviors among university students. This perspective moves beyond analyses focused on isolated variables and recognizes that physical activity, nutrition, stress management, and self-care often occur as interconnected behavioral patterns.

However, the association between lifestyle profiles and body phenotypes was weak. Students with normal body composition, hidden adiposity, and apparent adiposity were present in all three clusters. This indicates that a more favorable behavioral profile does not necessarily determine a specific body composition.

Body composition reflects cumulative processes and depends on factors not directly measured by the HPLP-II, including genetics, growth history, prior training, energy balance, sleep, sedentary behavior, hormonal factors, dietary intake, medication use, and socioeconomic context. Therefore, the profiles may guide group-level interventions but should not be used as diagnostic instruments or individual predictors.

It should also be noted that the clustering variables were limited to the six HPLP-II domains, standardized before the k-means procedure, and did not include the overall 52-item HPLP-II mean score, which is mathematically derived from these same domains and was therefore excluded to avoid redundancy. Nevertheless, cluster stability was not formally assessed in an independent sample. The clusters should therefore be interpreted as an exploratory solution and confirmed in future studies through cross-validation, stability analysis, and methods such as latent class or latent profile analysis.

### 4.7. Performance and Limitations of the Multivariable Model

The logistic regression model was statistically significant, showed acceptable fit, and explained 22.6% of the variability in hidden adiposity according to Nagelkerke’s R^2^. However, the model’s sensitivity was only 27.1%. In other words, it correctly identified fewer than one-third of participants with the phenotype.

The overall accuracy of 73.4% may be misleading when the outcome is less frequent than its absence. The model correctly classified 95.2% of participants without hidden adiposity but failed to identify most positive cases. It should therefore not be proposed as an individual screening tool.

This result reinforces the central argument of the article: sociodemographic characteristics and self-reported lifestyle data provide relevant information, but they do not replace body-composition assessment. Future studies should include variables with greater explanatory potential, such as waist circumference, training level, muscle strength, sleep, sedentary behavior, dietary intake, and cardiometabolic markers.

### 4.8. Scope and Limitations of Student Health Datafication

The contribution of this study extends beyond the comparison between BMI and body fat percentage. The integration of body measurements and lifestyle responses made it possible to construct a multidimensional representation of student health. This procedure may be understood as a bounded form of datafication, insofar as bodily and behavioral characteristics were transformed into data that could be classified, compared, and clustered [17,18]. However, the study did not examine an institutional digital health platform, a permanent monitoring system, or a broader process of digital transformation within the university.

The integrated analysis made it possible to identify cases of adiposity not detected by BMI, distinguish body phenotypes, and explore behavioral profiles. These findings show that the selection of indicators modifies what can be made visible through data. Nevertheless, a more detailed representation does not amount to a complete understanding of each student. The correlations were small, the association between lifestyle profiles and body phenotypes was weak, and the multivariable model lacked sufficient sensitivity for individual use.

The digital quantification of health may provide useful information for prevention and the planning of collective support, but it may also promote reductionism when indicators are interpreted outside their methodological and social contexts; the body should not be reduced to a single score or used as a mechanism of academic classification. Because the present study did not examine an institutional digital platform, continuous monitoring, or algorithm-driven decision-making, these governance considerations are noted here only as a bounded interpretive frame rather than as an empirical finding. Any future institutional use of these types of data should nonetheless be grounded in informed consent, data minimization, and non-stigmatizing interpretation, and should not be used to monitor, label, rank, or sanction students. In the present study, the predominance of male participants and the limited sensitivity of the model preclude any justification for automated applications or individual decisions based on its results.

### 4.9. Implications for Higher Education Institutions

The results do not justify the mandatory or indiscriminate implementation of body measurements. However, they support the development of voluntary health-assessment and health-promotion programs that complement BMI with information on body composition, physical activity, and well-being.

Universities could implement interventions that include:

1. Accessible and non-competitive opportunities for physical activity;
2. Education regarding the differences between body weight, muscle mass, and body fat;
3. Stress-management programs adapted to the academic calendar;
4. Evidence-based nutritional counseling;
5. Voluntary and confidential monitoring of health indicators;
6. Clinical referral when findings require professional assessment.

University interventions combining physical activity, nutrition, and healthy weight management may produce favorable outcomes, although their effectiveness depends on duration, participation, and methodological quality. Therefore, an isolated InBody assessment does not constitute an intervention. Measurement acquires preventive value only when accompanied by professional interpretation, education, and realistic opportunities for behavioral change.

Institutional communication should avoid classifications that reduce students to their bodily characteristics and should promote supportive conditions that strengthen belonging and reduce experiences of exclusion or discrimination [2].

### 4.10. Strengths

An important strength was the integration of two types of information obtained directly from participants: body composition estimated using the InBody 270 and lifestyles assessed using the HPLP-II. The availability of 303 complete cases allowed descriptive analyses, non-parametric comparisons, correlations, logistic regression, and exploratory segmentation to be conducted.

The study evaluated the classification performance of BMI through sensitivity, specificity, predictive values, and false-negative rate. This approach made it possible to identify the type of error produced by the indicator rather than merely demonstrating an association between BMI and body fat.

Both muscular and adipose components were also analyzed. This approach provided a more comprehensive assessment than the conventional classification of underweight, normal weight, overweight, or obesity. The use of the term hidden adiposity avoided attributing a clinical diagnosis that had not been assessed.

Another strength was the distinction between statistically significant associations and predictive utility. Although the regression model was statistically significant, its sensitivity was recognized as insufficient. Similarly, although the clusters were associated with body phenotypes, the magnitude of this association was acknowledged to be weak.

### 4.11. Limitations

The cross-sectional design constitutes the principal limitation. It is not possible to determine whether behaviors preceded body composition, whether body composition influenced particular behaviors, or whether both domains were shaped by common factors.

Convenience sampling and the inclusion of only two degree programs related to physical activity limit generalizability. The results do not necessarily represent all students at Universidad Técnica del Norte, students from other academic fields, or the broader Ecuadorian university population.

The distribution by sex was unequal. The predominance of male participants reduced the precision of estimates for women and may have affected comparisons among phenotypes. Furthermore, the interaction between sex and degree program was not assessed.

Bioelectrical impedance analysis provides indirect estimates and may be influenced by hydration status, recent food intake, exercise, and other prior conditions [14,16]. The InBody 270 is not equivalent to dual-energy X-ray absorptiometry and does not constitute a clinical diagnostic reference standard. Preparation conditions that were not individually controlled may have introduced variability.

The body fat cutoff points were used as operational criteria. Their specific validity among Ecuadorian university students was not examined. Classification may vary when different thresholds, reference methods, or criteria adjusted for age and population characteristics are applied.

Waist circumference, waist-to-height ratio, blood pressure, glucose, lipid profile, liver function, and inflammatory markers were not measured. Consequently, it cannot be determined whether hidden adiposity was accompanied by metabolic abnormalities.

The HPLP-II is a self-report instrument and may be subject to social desirability bias, recall errors, and differences in interpretation. The internal-consistency estimates obtained in this study do not constitute a comprehensive psychometric validation of the adapted 52-item form in Ecuadorian university students.

Finally, the clusters and regression model were not evaluated in an independent sample. Their results should be considered exploratory and should not be used to make individual predictions or automated institutional decisions.

### 4.12. Future Research Directions

Future studies should include students from different faculties, universities, and regions, with a more balanced distribution by sex. Longitudinal designs would allow changes to be observed throughout the semester, during examination periods, and across transitions between academic levels.

Body-composition assessment should be complemented by waist circumference, waist-to-height ratio, muscle strength, cardiorespiratory fitness, and metabolic markers. In a subsample, bioelectrical impedance analysis should be compared with dual-energy X-ray absorptiometry or another reference method.

Physical activity and sedentary behavior should be measured using accelerometry or validated devices, whereas sleep could be assessed with specific instruments. Dietary intake should be evaluated through food-frequency questionnaires, 24-h dietary recalls, or food records.

The adapted 52-item HPLP-II form used in the present study should be further evaluated among Ecuadorian university students through confirmatory factor analysis, assessment of convergent and discriminant validity, and measurement-invariance testing by sex and degree program.

Research on datafication should incorporate participatory methodologies. Students should be involved in defining which data are collected, communicating the results, and designing interventions. The objective should be to improve collective well-being without generating surveillance, stigma, or discrimination.

## 5. Conclusions

The integration of digital body-composition measurements and lifestyle data demonstrated how a bounded form of datafication can broaden the analytical representation of student health in higher education. By complementing body mass index with information on adiposity, muscular components, and health-promoting behaviors, differences that remained concealed under a classification based exclusively on weight and height became visible. This finding shows that the selection of indicators and measurement technologies is not neutral, as it determines which student characteristics can be identified and which institutional interventions may be proposed on that basis.

Elevated body fat percentage was identified in 53.5% of participants, while hidden adiposity was identified in 19.5% of the total sample and 32.1% of those with a normal BMI. Relative to the operational BIA-based criterion, BMI showed 63.6% classification agreement for elevated body fat and a false-negative rate of 36.4%, confirming that its use in isolation may produce an incomplete representation of body composition. Participants with hidden adiposity had greater adiposity, less favorable muscle-related indicators, and lower scores for physical activity, health responsibility, and stress management.

However, the availability of more data did not enable accurate prediction of individual body phenotypes. The overall 52-item HPLP-II mean score was not independently associated with hidden adiposity, the multivariable model showed low sensitivity, and the relationship between behavioral profiles and body phenotypes was weak. Therefore, these results do not justify using algorithms, lifestyle profiles, or body measurements to automatically classify students, establish diagnoses, or support individual academic decisions.

At the institutional level, the findings support voluntary health-promotion and preventive-monitoring programs integrating professional guidance, accessible physical activity, nutrition education, and stress management. The use of bodily and behavioral data should be grounded in informed consent, confidentiality, data minimization, purpose limitation, non-stigmatizing interpretation, and student participation in the design and communication of interventions. Datafication acquires social value when it contributes to collective well-being and the equitable allocation of support, rather than becoming a mechanism of surveillance or ranking.

Future longitudinal, multicenter, and participatory studies should include more diverse academic populations, objective measures of physical activity, cardiometabolic indicators, and reference methods for assessing body composition. It will also be necessary to examine how students perceive these technologies, the degree of trust they place in the institutions managing their data, and the conditions under which they consider their use in university health programs legitimate.

## Figures and Tables

**Table 1:** Data-cleaning, coding, and operational variable-definition criteria.

Criterion	Applied Decision
Unit of analysis	University student with a valid research-specific alphanumeric code recorded in both the InBody 270 and HPLP-II datasets.
Integrated dataset	A total of 303 students with complete and valid information from both assessments.
Duplicate-record rule	Only one record per participant was retained. For the InBody 270, the most recent valid measurement was kept; for the HPLP-II, the first complete and valid response was retained.
Participant code	An alphanumeric code composed of the abbreviation of the degree program, the academic level, and a sequential number assigned to each participant.
AF	Physical Activity and Sport Pedagogy degree program.
ED	Sports Training degree program.
Cutoff for elevated BMI	BMI ≥ 25.0 kg/m^2^.
Cutoff for elevated body fat	Body fat percentage > 20% in men and >28% in women, according to the sex-specific operational criteria used in this study.
Primary phenotype	Hidden adiposity: normal BMI combined with an elevated body fat percentage according to sex.

Note. BMI: body mass index; HPLP-II: Health-Promoting Lifestyle Profile II. The applied criteria ensured that each participant contributed only one valid record to the integrated dataset. Hidden adiposity was considered an operational body-composition phenotype rather than a clinical diagnosis.

**Table 2:** Sociodemographic characteristics, body composition, and body phenotypes of the sample. (**A**) Sociodemographic and categorical variables. (**B**) Continuous variables.

(**A**)				
**Variable**	**Category**		**Frequency, *n***	**Percentage, %**
Sex	Female		64	21.1
	Male		239	78.9
Degree program	Physical Activity and Sport Pedagogy		160	52.8
	Sports Training		143	47.2
BMI classification	Underweight		5	1.7
	Normal BMI		184	60.7
	Elevated BMI		114	37.6
Body fat classification	Normal body fat		141	46.5
	Elevated body fat		162	53.5
Body phenotype	Underweight		5	1.7
	Normal BMI and normal body fat		125	41.3
	Hidden adiposity		59	19.5
	Elevated BMI and normal body fat		11	3.6
	Apparent adiposity		103	34.0
(**B**)				
**Variable**	***N***	**Mean**	**SD**	**Median [P25–P75]**
Age, years	303	21.55	2.41	21.00 [20.00–23.00]
Body mass index, kg/m^2^	303	24.18	3.23	23.70 [21.85–26.15]
Body fat percentage	303	22.85	8.15	21.90 [16.15–27.85]
Skeletal muscle mass, kg	303	28.76	5.20	28.90 [26.00–32.10]
Visceral fat level	303	5.75	3.42	5.00 [3.00–7.00]
Skeletal muscle index	303	7.63	0.88	7.70 [7.10–8.20]
Fat-free mass index, kg/m^2^	303	18.49	1.89	18.60 [17.20–19.70]

Note. Categorical variables are presented as frequencies and percentages, whereas continuous variables are reported as means, standard deviations, and medians with the 25th and 75th percentiles. SD: standard deviation; BMI: body mass index; P25: 25th percentile; P75: 75th percentile. Elevated BMI was defined as a value ≥ 25.0 kg/m^2^. Hidden adiposity was defined as the simultaneous presence of a normal BMI and an elevated body fat percentage according to sex. Apparent adiposity was defined as elevated BMI accompanied by elevated body fat.

**Table 3:** Descriptive statistics and internal consistency of the HPLP-II.

Overall 52-Item Composite	*N*	Number of Items	Mean	SD	Median [P25–P75]	Cronbach’s α
Overall 52-item HPLP-II mean	303	52	2.53	0.39	2.50 [2.29–2.76]	0.941
Physical activity	303	9	2.38	0.58	2.33 [2.00–2.78]	0.854
Nutrition	303	10	2.50	0.45	2.40 [2.20–2.80]	0.766
Health responsibility	303	7	2.16	0.50	2.14 [1.86–2.43]	0.805
Stress management	303	8	2.43	0.47	2.38 [2.13–2.75]	0.741
Interpersonal relations	303	9	2.65	0.45	2.67 [2.33–3.00]	0.787
Positive psychology	303	9	2.99	0.51	3.00 [2.67–3.39]	0.851

Note. HPLP-II scores range from 1 to 4, with higher values indicating a greater frequency of health-promoting behaviors. SD: standard deviation; HPLP-II: Health-Promoting Lifestyle Profile II; P25: 25th percentile; P75: 75th percentile.

**Table 4:** Agreement and classification performance of BMI for identifying elevated body fat. (**A**) Cross-classification of BMI and body fat. (**B**) Association and agreement between the classification criteria. (**C**) Classification performance of BMI.

(**A**)			
**BMI Classification**	**Normal Body Fat**	**Elevated Body Fat**	**Total**
Non-elevated BMI	130	59	189
Elevated BMI	11	103	114
Total	141	162	303
(**B**)			
**Indicator**	**Value**		***p***
Pearson’s chi-square	99.94		<0.001
φ	0.574		<0.001
Cramer’s V	0.574		<0.001
Cohen’s κ	0.546		—
(**C**)			
**Indicator**		**Percentage**	
Sensitivity		63.6	
Specificity		92.2	
Positive predictive value		90.4	
Negative predictive value		68.8	
false-negative rate		36.4	
Overall accuracy		76.9	

Note. BMI: body mass index. Elevated BMI was defined as a value ≥ 25.0 kg/m^2^. Elevated body fat estimated by bioelectrical impedance analysis was used as an operational comparison criterion rather than as a clinical diagnostic reference standard. Sensitivity represents the ability of BMI to identify participants with elevated body fat, whereas specificity represents its ability to correctly identify those with normal body fat.

**Table 5:** Comparison of HPLP-II scores and body-composition indicators between students with normal BMI and normal body fat and those with hidden adiposity. (**A**) Health-promoting lifestyle domains. (**B**) Body-composition indicators.

(**A**)					
**Variable**	**Normal BMI and Normal Body Fat, *n* = 125 Mean (SD); Median**	**Hidden Adiposity, *n* = 59 Mean (SD); Median**	**U**	***p***	**r**
Overall HPLP-II	2.56 (0.40); 2.56	2.44 (0.35); 2.44	4283.5	0.077	0.130
Physical activity	2.48 (0.60); 2.33	2.16 (0.48); 2.11	4842.0	<0.001	0.253
Nutrition	2.50 (0.47); 2.40	2.39 (0.37); 2.40	4142.5	0.176	0.100
Health responsibility	2.19 (0.50); 2.14	2.05 (0.46); 2.00	4417.0	0.030	0.160
Stress management	2.47 (0.47); 2.50	2.32 (0.46); 2.25	4414.5	0.031	0.159
Interpersonal relations	2.63 (0.46); 2.56	2.69 (0.45); 2.67	3325.5	0.282	0.079
Positive psychology	3.01 (0.50); 3.00	2.91 (0.58); 3.00	4052.0	0.279	0.080
(**B**)					
**Variable**	**Normal BMI and Normal Body Fat, *n* = 125 Mean (SD); Median**	**Hidden Adiposity, *n* = 59 Mean (SD); Median**	**U**	***p***	**r**
Skeletal muscle mass, kg	29.14 (3.92); 28.90	24.80 (4.48); 25.80	5553.5	<0.001	0.408
Fat-free mass index, kg/m^2^	18.29 (1.23); 18.40	16.94 (1.59); 17.20	5433.0	<0.001	0.382
Skeletal muscle index	7.60 (0.60); 7.60	6.88 (0.80); 7.00	5539.5	<0.001	0.405
Fat mass index, kg/m^2^	3.54 (0.92); 3.40	6.29 (1.17); 6.30	190.0	<0.001	0.765
Visceral fat level	3.03 (1.06); 3.00	6.25 (1.49); 6.00	200.5	<0.001	0.774
Body fat percentage, %	16.14 (3.78); 15.70	27.10 (5.06); 26.50	225.5	<0.001	0.757

Note. Descriptive data are presented as means, standard deviations, and medians. Between-group differences were evaluated using the Mann–Whitney U test. SD: standard deviation; HPLP-II: Health-Promoting Lifestyle Profile II; BMI: body mass index; r: effect size. Hidden adiposity was defined as the coexistence of a normal BMI and an elevated body fat percentage according to sex. Values of *p* < 0.05 were considered statistically significant.

**Table 6:** Comparison of HPLP-II scores and body-composition indicators across body phenotypes. (**A**) Health-promoting lifestyle domains. (**B**) Body-composition indicators.

(**A**)					
**Variable**	***N***	**Kruskal–Wallis H**	**df**	***p***	**ε^2^**
Overall HPLP-II	298	11.33	3	0.010	0.028
Physical activity	298	22.82	3	<0.001	0.067
Nutrition	298	7.44	3	0.059	0.015
Health responsibility	298	12.63	3	0.006	0.033
Stress management	298	8.10	3	0.044	0.017
Interpersonal relations	298	3.22	3	0.359	0.001
Positive psychology	298	5.96	3	0.113	0.010
(**B**)					
**Variable**	***N***	**Kruskal–Wallis H**	**df**	***p***	**ε^2^**
Skeletal muscle mass	298	60.01	3	<0.001	0.194
Fat-free mass index	298	95.59	3	<0.001	0.315
Skeletal muscle index	298	89.16	3	<0.001	0.293
Fat mass index	298	222.60	3	<0.001	0.747
Visceral fat level	298	225.99	3	<0.001	0.758
Body fat percentage, %	298	201.04	3	<0.001	0.674

Note. The four phenotypes included were normal BMI and normal body fat, hidden adiposity, elevated BMI with normal body fat, and apparent adiposity. The five participants classified as underweight were excluded. H: Kruskal–Wallis statistic; df: degrees of freedom; ε^2^: epsilon-squared. A value of *p* < 0.05 was considered statistically significant.

**Table 7:** Statistically significant Spearman’s rank correlations between HPLP-II scores and body-composition indicators.

HPLP-II Variable	Body-Composition Indicator	*N*	Spearman’s ρ	*p*
Overall HPLP-II	Skeletal muscle mass	303	0.229	<0.001
	Skeletal muscle index	303	0.204	<0.001
	Fat-free mass index	303	0.186	0.001
	Body fat percentage	303	−0.143	0.012
Physical activity	Skeletal muscle index	303	0.229	<0.001
	Fat-free mass index	303	0.228	<0.001
	Skeletal muscle mass	303	0.227	<0.001
	Body fat percentage	303	−0.174	0.002
	Fat mass index	303	−0.120	0.037
Health responsibility	Skeletal muscle mass	303	0.218	<0.001
	Skeletal muscle index	303	0.190	<0.001
	Fat-free mass index	303	0.173	0.002
	Body fat percentage	303	−0.149	0.009
Stress management	Skeletal muscle mass	303	0.199	<0.001
	Skeletal muscle index	303	0.171	0.003
	Fat-free mass index	303	0.159	0.006
	Body fat percentage	303	−0.140	0.014
Nutrition	Skeletal muscle mass	303	0.197	<0.001
	Skeletal muscle index	303	0.169	0.003
	Fat-free mass index	303	0.169	0.003
Positive psychology	Skeletal muscle mass	303	0.189	<0.001
	Skeletal muscle index	303	0.164	0.004
	Fat-free mass index	303	0.135	0.018

Note. Only statistically significant correlations identified in the analysis are presented. HPLP-II: Health-Promoting Lifestyle Profile II. Positive values indicate that higher HPLP-II scores were associated with higher values of the corresponding body-composition indicator, whereas negative values indicate an inverse association. Spearman’s rho does not imply causality.

**Table 8:** Binary logistic regression of factors associated with hidden adiposity among participants with normal BMI.

Predictor Variable	B	Standard Error	Wald	OR	95% CI for OR	*p*
Female sex	1.635	0.426	14.722	5.130	2.225–11.827	<0.001
Sports Training degree program	−1.268	0.375	11.400	0.281	0.135–0.588	<0.001
Age	0.030	0.073	0.164	1.030	0.893–1.189	0.686
Overall HPLP-II	−0.629	0.470	1.785	0.533	0.212–1.341	0.182

Note. The dependent variable was coded as hidden adiposity = 1 and normal BMI with normal body fat = 0. The reference categories were male sex and enrollment in the Physical Activity and Sport Pedagogy degree program. B: regression coefficient; 95% CI: 95% confidence interval; BMI: body mass index; HPLP-II: Health-Promoting Lifestyle Profile II; OR: odds ratio. Model fit: χ^2^(4) = 32.39, *p* < 0.001; Cox and Snell R^2^ = 0.161; Nagelkerke R^2^ = 0.226; Hosmer–Lemeshow test: χ^2^(8) = 5.93, *p* = 0.655. Overall accuracy = 73.4%; sensitivity = 27.1%; specificity = 95.2%.

**Table 9:** Health-promoting lifestyle profiles and distribution of body phenotypes. (**A**) Final cluster centers. (**B**) Distribution of body phenotypes according to lifestyle profile.

(**A**)					
**Variable**	**Profile 1: Less Favorable (*n* = 118)**		**Profile 2: Intermediate (*n* = 131)**	**Profile 3: More Favorable (*n* = 54)**	
Physical activity	1.99		2.44	3.11	
Nutrition	2.23		2.53	3.02	
Health responsibility	1.85		2.16	2.82	
Stress management	2.05		2.53	2.98	
Interpersonal relations	2.37		2.71	3.13	
Positive psychology	2.59		3.12	3.49	
(**B**)					
**Lifestyle Profile**	**Normal BMI and Normal Body Fat**	**Hidden Adiposity**	**Elevated BMI and Normal Body Fat**	**Apparent Adiposity**	**Total**
Profile 1: Less favorable	39	26	1	48	114
Profile 2: Intermediate	63	26	6	36	131
Profile 3: More favorable	23	7	4	19	53
Total	125	59	11	103	298

Note. Cluster analysis was performed using the six HPLP-II domains (physical activity, nutrition, health responsibility, stress management, interpersonal relations, and positive psychology); the overall 52-item HPLP-II score was excluded from the clustering variables to avoid redundancy with its constituent domains and is reported in the text as a descriptive summary only. The labels assigned to the profiles are descriptive and do not represent clinical categories. Five participants classified as underweight were excluded from the comparison with body phenotypes; therefore, the totals in Panel B differ from the original cluster sizes. HPLP-II: Health-Promoting Lifestyle Profile II; BMI: body mass index. Association between profiles and phenotypes: χ^2^(6) = 14.87, *p* = 0.021; Cramer’s V = 0.158. The ANOVA table generated by the k-means procedure was interpreted solely for descriptive purposes and not as an independent inferential test.

## Data Availability

The data supporting the findings of this study are available from the corresponding author upon reasonable request. The dataset is not publicly available because it contains sensitive information on body composition and lifestyle, and the informed consent obtained from participants did not include the public deposition of individual-level data. Any access to the data will be subject to appropriate anonymization procedures and the corresponding institutional and ethical considerations.
